# Fecal Bile Acids in Canine Chronic Liver Disease: Results from 46 Dogs

**DOI:** 10.3390/ani14213051

**Published:** 2024-10-22

**Authors:** Verena Habermaass, Francesco Bartoli, Eleonora Gori, Rebecca Dini, Aurora Cogozzo, Caterina Puccinelli, Alessio Pierini, Veronica Marchetti

**Affiliations:** 1 Department of Veterinary Sciences, Veterinary Teaching Hospital “Mario Modenato”, University of Pisa, Via Livornese Lato Monte, San Piero a Grado, 56122 Pisa, Italy; verena.habermaass@phd.unipi.it (V.H.); veronica.marchetti@unipi.it (V.M.); 2 Department of Translational Research and New Technologies in Medicine and Surgery, University of Pisa, Via Savi 10, 56126 Pisa, Italy

**Keywords:** canine microbiome, gut–liver axis, fecal metabolome, canine liver disease, bile acid diarrhea, canine biliary tract disease, canine cholestasis

## Abstract

The concentrations of fecal and serum bile acids (BAs) are known to be altered in human patients with chronic liver diseases (CLDs), especially those with biliary tract involvement (BTD). Scarce literature is available regarding fecal BA modifications during canine CLDs. This study aimed to evaluate fecal BAs in canine CLDs according to different clinical and clinicopathological variables. Forty-six dogs were enrolled. Canine feces were analyzed by HPLC. Cholic Acid (CA), Chenodeoxycholic Acid (CDCA), Ursodeoxycholic Acid (UDCA), Deoxycholic Acid (DCA), and Lithocholic Acid (LCA) were measured, and primary BAs (CA + CDCA), secondary BAs (UDCA + DCA + LCA), and the primary/secondary (P/S) ratio were calculated. Primary BAs (*p* < 0.0001), CA (*p* = 0.0003), CDCA (*p* = 0.003), the P/S ratio (*p* = 0.002), and total BAs (*p* = 0.005) were significatively higher in BTD dogs (n = 18) compared to in non-BTD dogs (n = 28). Fecal secondary BAs did not statistically differ between BTD and non-BTD dogs. Gastrointestinal clinical signs (*p* = 0.028) and diarrhea (*p* = 0.03) were significantly more prevalent in BTD dogs compared to in non-BTD dogs, supporting the hypothesis of some pathological mechanisms assimilable to bile acid diarrhea (BAD). Our results could reflect imbalances of the fecal BA metabolism in dogs with CLDs. Further studies involving gut microbiome and metabolomic assessment are needed to better understand the possible clinical implications of BA metabolism disruption and their potential role in canine CLDs.

## Simple Summary

Since bile acids (BAs) represent one of the metabolic joining links between the gut and the liver, fecal concentrations of BAs could be altered in human patients with different kinds of chronic liver diseases (CLDs). In veterinary medicine, scarce literature is available regarding their potential modification during canine CLDs. The present study aimed to evaluate fecal BAs in dogs with different kinds of CLDs, especially differentiating between dogs with CLDs with and without biliary tract involvement (BTD vs non-BTD). Forty-six dogs were enrolled in this study. Canine feces were analyzed for the quantification of Cholic Acid (CA), Chenodeoxycholic Acid (CDCA), Ursodeoxycholic Acid (UDCA), Deoxycholic Acid (DCA), Lithocholic Acid (LCA), Primary BAs (CA + CDCA), secondary BAs (UDCA + DCA + LCA), and the primary/secondary (P/S) BA ratio. Primary BAs, CA, CDCA, the P/S ratio, and total BAs were significatively higher in BTD dogs (n = 18) compared to in non-BTD dogs (n = 28). Secondary BAs, UDCA, DCA, and LCA did not differ significantly between dogs with and without BTD. Gastrointestinal clinical signs were significantly more prevalent in BTD dogs compared to in non-BTD dogs. Our results agree with some evidence in human CLDs and could reflect imbalances in the liver–gut interaction and liver function impairment.

## 1. Introduction

Bile acids (BAs) are amphipathic molecules derived from cholesterol in the liver that play essential roles in digestion, the absorption of dietary fats, and the regulation of cholesterol levels. They are synthesized in hepatocytes through a multi-step process involving several enzymes. Additionally, bile acids act as signaling molecules through nuclear receptors like the farnesoid X receptor (FXR) and G protein-coupled bile acid receptor (GPBAR1 or TGR5), modulating lipid, glucose, and energy metabolism, as well as inflammatory responses [1,2].

In humans, fecal BAs play a crucial role in chronic liver diseases (CLDs). In human CLD, impaired liver function leads to alterations in the metabolism of BA’s, resulting in an alteration of their composition and concentration in the gastrointestinal tract [3]. In CLD, intrahepatic cholestasis, bile duct obstruction, or hepatocellular dysfunction can disrupt normal bile acid synthesis and secretion [3]. Additionally, changes in gut microbiota composition, which can often be observed in CLD, can further modulate the metabolism of BAs through enzymatic reactions. Several studies have demonstrated the potential of fecal BAs as diagnostic and prognostic markers in CLD [4,5]. For instance, elevated levels of fecal BAs have been documented in various CLDs in human patients, including primary biliary cholangitis (PBC), primary sclerosing cholangitis (PSC), and non-alcoholic fatty liver disease (NAFLD). Elevated levels of specific primary BAs, such as Cholic Acid (CA) and Chenodeoxycholic Acid (CDCA), have been associated with disease severity and progression in PBC and PSC [6,7]. Moreover, fecal BA profiling has shown promise in predicting the response to therapy and monitoring treatment efficacy in CLD patients [8].

The veterinary literature is currently mainly focused on the alteration of fecal BAs in canine chronic enteropathy [9,10], but scarce literature is available regarding their potential modifications during CLDs. In canine CLD, various conditions, such as chronic hepatitis, hepatic lipidosis, and portosystemic shunts, could result in hepatocellular dysfunction or intrahepatic cholestasis [11,12,13]. These hepatic abnormalities could lead to the intrahepatic accumulation of bile acids [11,13]. Additionally, changes in gut microbiota composition, which have recently been observed in dogs with CLD [14], can further influence the metabolism of bile acids through enzymatic modifications. As in humans, dogs with CLD could have bile acid metabolism abnormalities due to impaired hepatic function, leading to alterations in the composition and concentration of BAs in the gastrointestinal tract. Studying the complex relationship between hepatic function, gut microbiota, and the metabolism of BAs in dogs may help to improve the management and outcomes of CLD, providing insights into disease pathogenesis and potential therapeutic targets. Thus, the present study aimed to evaluate fecal BA patterns in dogs with CLDs, according to different clinical and clinicopathological variables. We suppose that fecal BAs may be altered in dogs with CLD through different possible pathogenetical mechanisms, potentially involving alterations in the liver–gut interaction and homeostasis, and thus possibly associated with gastrointestinal signs.

## 2. Materials and Methods

**Figure 1:**
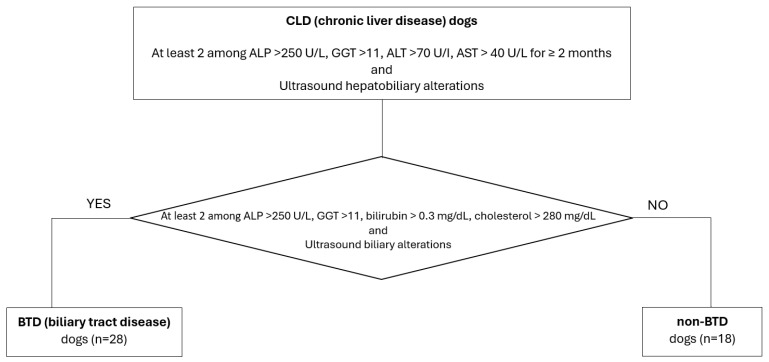
Flowchart of the study population classification, according to CLD and the BTD/non-BTD subgroups.

This study was conducted in accordance with the Declaration of Helsinki and approved by the Ethics Committee of the University of Pisa (protocol code n. 41, date of approval: 29 October 2020). Client-owned dogs referred to the Internal Medicine Service of the Veterinary Teaching Hospital of the University of Pisa between January 2020 and January 2023 with a diagnosis of chronic liver disease (CLD) were included. A CLD diagnosis was based on the dog’s medical history, a physical examination, hematology, and blood biochemistry, as well as abdominal ultrasonography. Dogs were prospectively recruited, and the diagnosis of CLD was based on the concurrent presence of persistent increased liver enzymes (>2 months), specifically at least 2 among alkaline phosphatase (ALP) >250 U/L (reference range 45–250 U/L), gamma-glutamyl transferase (GGT) >11 (reference range 2–11 U/L), alanine aminotransferase (ALT) >70 U/I (reference range 20–70 U/L), and aspartate aminotransferase (AST) >40 U/L (reference range 15–40 U/L); ultrasound chronic hepatobiliary alterations, such as hyperechoic parenchyma, abnormalities in the hepatic dimensions and/or margins; the presence of nodular hepatic lesions, referred to as benign hyperplasia; thickened, hyperechoic and irregular gallbladder walls; abnormalities in the gallbladder contents (e.g., mucocele, non-gravity-dependent biliary sludge, cholelithiasis); a chronic intrahepatic biliary tree or common biliary duct dilatation; and mineralization of the intrahepatic biliary tree. Biliary tract disease (BTD) was identified in the case of two or more of the following laboratory alterations: ALP >250 U/L (reference range 45–250 U/L), GGT >11 U/L (reference range 2–11 U/L), total bilirubin >0.3 mg/dL (reference range 0.07–0.3 mg/dL), cholesterol >280 mg/dL (reference range 120–280 mg/dL), and one or more concurrent ultrasonographic biliary tract alterations. According to this classification, the enrolled CLD dogs were divided into subgroups according to biliary tract involvement, specifically BTD and non-BTD (Figure 1). Clinical information, such as ongoing diet and therapies, was registered. Dogs that had received antibiotics, UDCA, probiotics, or synbiotic treatment in the previous 2 months were excluded from the study. All dogs with a history of chronic enteropathy, or with an abdominal ultrasound showing intestinal alterations, were also excluded.

For each dog, clinical history, copro-parasitological evaluation, complete hematobiochemical profile, and abdominal ultrasound were assessed. Dogs presenting other significative comorbidities (renal, cardiac, hematologic, pancreatic diseases, esocrine pancreatic insufficiency, or significative primary enteropathies) or who tested positive for intestinal parasites were excluded. At the time of enrollment, clinical history information was registered. Information about eventual mild gastrointestinal signs (episodes of vomiting and/or diarrhea) were collected. Dogs with CLD secondary to endocrine disorder were not excluded from the present study.

### 2.1. Fecal Bile Acids Analysis

Fecal samples from the dogs were stored at −80 °C, lyophilized, and then subjected to extraction and analysis through High-Performance Liquid Chromatography (HPLC). The concentrations of CA, CDCA, UDCA, DCA, and LCA were determined (Sigma-Aldrich St. Louis, MO, USA, C1129, D2510, L6250, D2510, U5127). Calculations included primary bile acids (CA + CDCA), secondary bile acids (UDCA + DCA + LCA), the ratio of primary to secondary bile acids, and total bile acids (sum of the primary and secondary bile acids). CA, CDCA, UDCA, DCA, and LCA were from Sigma (Sigma-Aldrich St. Louis, MO, USA). The sample preparation and HPLC analytical procedures were adapted from a previously established method by Kakiyama et al. [15], with specific modifications. Approximately 5–10 mg of lyophilized feces was ground into a fine powder prior to analysis. The powdered sample was suspended in 250 µL of cold water and heated to 90 °C in a tightly sealed tube for 10 min. After heating, the sample was visually inspected, and any remaining large particles were further disintegrated by placing the sample in an ultrasonic bath (Bandelin RK 255 H, capacity 5.7l, Bandelin electronic GmbH & Co., Berlin, Germany) filled with distilled water and sonicating it until complete particle disruption was achieved. The suspension was then mixed with 250 µL of a sodium acetate buffer solution (100 mM, pH 5.6), which contained 15 units of cholylglycine hydrolase (Sigma-Aldrich C4018) and 150 units of sulfatase (Sigma-Aldrich, S9626), and incubated at 37 °C for 16 h. To terminate the reaction, 500 µL of isopropanol and 100 µL of 1 N NaOH were added, followed by heating at 60 °C for two h. After this, an internal standard (IS), consisting of 50 nmol of norDCA (Avanti Polar Lipids, Alabama, USA, 700240P) and 3 mL of 0.1 N NaOH, was introduced. Bile acids were then extracted from the fecal matrix via ultrasonication at room temperature for an hour. After centrifuging the mixture at 15,000× *g* for 20 min (Beckman Coulter s.r.l Milan, Italy, Allegra), the supernatant was completely aspirated and collected, and the remaining pellet was rinsed with 2 mL of 0.1 N NaOH. The extracts were combined and processed using a Waters Sep-Pak tC18 cartridge (Waters, Milan, Italy), 500 mg sorbent, WAT043395) and pre-conditioned with 10 mL each of methanol and water. The cartridge was sequentially rinsed with 5 mL of water, 4 mL of 15% acetone, and another 5 mL of water. The fecal bile acids retained in the cartridge were then eluted with 6 mL of methanol and dried under a nitrogen stream at a temperature below 40 °C.

The extracted, unconjugated bile acids were derivatized into their 24-phenacyl esters. To achieve this, the dried extract was treated with 150 µL of 10 mg/mL TEA (Sigma-Aldrich, 471283) in acetone and 150 µL of 12 mg/mL phenacyl bromide (2-acetobromophenone, Supelco Sigma, Milan, Italy, 77450) in acetone. The mixture was subjected to ultrasonication and heated at 50 °C in a sealed glass tube for 1.5 h. After the reaction, the mixture was diluted with 2 mL of acetone and applied to a Waters Sep-Pak^®^ silica cartridge (Waters, 500 mg sorbent, WAT036950), which had been conditioned with 5 mL of acetone. The bile acid 24-phenacyl esters were then eluted from the column using 4 mL of acetone, and the resulting solution was dried under a nitrogen stream.

Finally, the residue was reconstituted in 200 µL of 82% methanol, filtered through a 0.45 µm filter, and 20 µL of the filtrate was injected into the HPLC system. The system comprised a Jasco separation module with a DAD detector, managed by Chrome Nav software (ChromNAV 2.0 HPLC Software). Separation was performed using a Phenomenex Luna (Phenomenex Inc., Bologna, Italy) C18 column (150 mm × 3 mm, particle size 3 µm) with a guard column (20 mm × 3 mm i.d.), maintained at 40 °C. The mobile phase was 82% methanol, and the flow rate was set to 0.7 mL/min. Detection of individual bile acid 24-phenacyl esters was carried out by monitoring their absorption at 254 nm. In referral to the HPLC analysis of fecal bile acids, since all targeted compounds are detected at 254 nm, the limit of quantification (LOQ) and limit of detection (LOD) and the retention time for each compound are provided in the Supplementary Materials.

### 2.2. Statistical Analysis

Statistical analysis was performed using the software GraphPad Prism, Version 9, San Diego, CA, USA 2020. Considering the non-normal distribution of variables according to the Kolmogorov–Smirnov normality test, the Mann–Whitney U test or Kruskal–Wallis test was applied to compare the fecal BAs (CA, CDCA, UDCA, DCA, LCA, primary, secondary, P/S ratio, and total fecal BAs) between the various subgroups of CLD dogs, classified according to different clinicopathological variables (the presence/absence of clinical gastrointestinal signs, hypercholesterolemia, and concurrent endocrinopathies). Chi-square tests or Fisher’s exact tests were used to compare the prevalence of gastrointestinal signs (vomiting and/or diarrhea) or diarrhea between the BTD and non-BTD dogs. Statistical significance was identified for *p* values < 0.05.

## 3. Results

### 3.1. Animals

Forty-six dogs were enrolled, with a median age of 10 years (2.5–14.7 years). In total, 23 out of 46 (50%) were female (13 neutered, 10 intact) and 23 (50%) were male (3 neutered, 20 intact). The majority of dogs were mixed breed (n = 13; 28%), followed by Poodle (n = 3; 6.5%), Dachshund (n = 3; 6.5%), Cocker spaniel (n = 3; 6.5%), Yorkshire terrier (n = 3; 6.5%), Maltese (n = 3; 6.5%), Cavalier King Charles Spaniel (n = 2; 4.3%), Golden Retriever (n = 2; 4.3%), Jack Russell Terrier (n = 2; 4.3%), Labrador Retriever (n = 2; 4.3%), Shih Tzu (n = 2; 4.3%), West Highland White Terrier (n = 2; 4.3%), Setter (n = 1; 2.2%), Zwergpinscher (n = 1; 2.2%), German Shepherd (n = 1; 2.2%), French bulldog (n = 1; 2.2%), Breton (n = 1; 2.2%), and Boxer (n = 1; 2.2%). The serum biochemical findings for CLD dogs are reported in Table 1. With respect to diet, the majority of dogs were fed a home-cooked or commercial maintenance diet (n = 22; 48%), followed by dogs fed a commercial hyperdigestible diet (n = 15; 33%), while three dogs (6%) were fed a commercial Diabetic diet and six (13%) a commercial hepatic diet.

At the moment of inclusion, 26 dogs (56%) had gastrointestinal clinical signs (vomiting and/or diarrhea); specifically, 17 dogs (37%) had diarrhea and 18 (39%) had vomiting. Twenty-eight dogs (61%) were diagnosed with BTD according to the criteria reported above. Considering biliary tract involvement, 20/28 (71%) of the BTD dogs and 7/18 (39%) of the non-BTD dogs were presenting clinical gastrointestinal signs at the time of enrollment. Similarly, 14/28 (50%) of the BTD dogs and 3/18 (16%) of the non-BTD dogs were presenting diarrhea. The prevalence of gastrointestinal clinical signs or diarrhea was significatively higher in the BTD dogs compared to in the non-BTD dogs (respectively, *p* = 0.028, *p* = 0.03). In total, 15 out of the 46 dogs (36%) were diagnosed with at least one endocrine disease: 4 dogs had diabetes mellitus, 1 dog had hypoadrenocorticism, 3 dogs had hyperadrenocorticism, 6 dogs had hypothyroidism, and 1 dog had concurrent hypothyroidism and hypoadrenocorticism.

### 3.2. Fecal Bile Acids

The global results of fecal BAs are reported in Table 2. Some significant differences were observed for fecal BAs according to the presence of biliary tract involvement, as reported in Table 2. Primary BAs (CA, CDCA), the P/S ratio, and total BAs were significatively higher in dogs with BTD (n = 18) compared to in dogs without significant biliary tract involvement (n = 28; respectively, *p* = < 0.0001, *p* = 0.002, *p* = 0.005). Conversely, secondary BAs (UDCA, DCA, LCA) did not differ significantly between dogs with and without BTD (*p* = 0.3).

No significant differences were found in CLD dogs for single fecal BAs, primary BAs, secondary BAs, P/S ratio, and total BAs in relation to serum cholesterol, gastrointestinal clinical signs, meaning vomiting and/or diarrhea, or endocrine disorders.

## 4. Discussion

Our results showed some alteration in fecal BAs according to biliary tract involvement. This may reflect an impairment of the metabolism of fecal BAs, resulting from the complex interaction between the gut, the liver, and gut microbiota (GM).

BAs are synthesized in the liver, from cholesterol, through a series of enzymatic reactions. Before being secreted into bile, the primary BAs (CA and CDCA) are conjugated with either glycine or taurine through conjugation that increases their solubility and reduces their toxicity [2]. Conjugated BAs are secreted into the bile, stored in the gallbladder, and released into the small intestine. In the small intestine, BAs emulsify dietary fats, facilitating the action of pancreatic lipase and aiding in the formation of micelles, which are essential for fat absorption [2]. Many BAs are reabsorbed in the ileum and transported back to the liver via the portal circulation, through a process known as enterohepatic circulation. Some primary BAs are converted to secondary BAs (DCA, UDCA, and LCA) by intestinal bacteria through deconjugation, dehydroxylation, and epimerization reactions [2]. The synthesis of BAs is tightly regulated by feedback inhibition. High levels of BAs in the liver inhibit CYP7A1 activity, thereby reducing bile acid synthesis. BAs activate the farnesoid X receptor (FXR), which regulates genes involved in the synthesis, transport, and enterohepatic circulation of BAs [2]. A small fraction of the BAs escape reabsorption and are excreted in the feces. This loss is compensated by the de novo synthesis of BAs in the liver [1,16,17].

Based on our results, the presence of biliary tract involvement seems to be the main cause of BA modifications observed in CLD dogs.

In humans, primary BAs are considered essential for various physiological processes in the gut; however, under certain conditions, they can also cause negative effects, such as the promotion of inflammation [18], the disruption of intestinal barrier function [19], intestinal dysbiosis [20], and colon carcinogenesis [21]. On the other hand, secondary BAs, formed through the microbial metabolism of primary BAs in the colon, can promote various positive effects on gut health, such as the development of anti-inflammatory properties [20], providing a regulatory function on the gut barrier [22], the modulation of GM [23], and offering potential anti-cancer effects [21]. Dogs with chronic enteropathy are known to have increased levels of primary BAs and decreased levels of secondary BAs [24]. In this study, the increase in fecal primary BAs (CA, CDCA) and total BAs in CLD dogs with biliary tract involvement is consistent with the trend observed in human patients with biliary tract disorders [25]. Our population of dogs with BTD showed higher primary and total levels of BAs, but no significative difference in the secondary BAs, compared to CLD dogs without biliary tract involvement. We may suppose that the metabolism and pathophysiology of these compounds may be different in dogs with chronic enteropathy compared to dogs with chronic liver disease. The metabolism of BAs during chronic liver disease and cholestatic liver disease is complex, since it is potentially related to both liver function impairment and eventual cholestatic processes. In human chronic cholestasis, which is characterized by impaired bile flow from the liver, fecal primary BAs are increased, due to several underlying mechanisms. One of the potential mechanisms is biliary obstruction. In fact, during cholestasis, there is a blockage or impairment of bile flow from the liver into the intestine. This obstruction can occur at various levels: within the bile ducts (e.g., due to gallstones or strictures) or within the liver (e.g., due to inflammation or fibrosis). The BAs that cannot be excreted via bile flow accumulate in the liver and spill over into the systemic circulation, ultimately leading to increased fecal excretion [26]. Another mechanism may be hepatic overproduction. In fact, in response to impaired bile flow, the liver may compensate by increasing the production of bile acids, which contributes to the elevated levels of fecal primary BAs [2]. It is important to underline that during biliary tract disease with cholestasis a reduced production of BAs is reported. In fact, the suppression of BA synthesis can be observed, and it is hypothesized to act as a protective mechanism against hepatic BA accumulation and cytotoxicity [27]. Enterohepatic circulation alterations can also cause an alteration in fecal primary BAs; indeed, cholestasis can interrupt the enterohepatic circulation, the process through which primary BAs are reabsorbed into the intestine and transported back to the liver. In this scenario, with an impaired bile flow, less primary BAs are reabsorbed, leading to an increase in their fecal excretion [28,29]. Finally, chronic liver disease is often associated with alterations in the composition of gut microbiota, which can affect the metabolism of BAs. Since intestinal bacteria can modify BAs through deconjugation and dehydroxylation, dysbiosis can lead to changes in the transformation and reabsorption of BAs, contributing to increased fecal levels of primary BAs [20,30,31].

Gastrointestinal signs and diarrhea were more frequent in BTD dogs. This may be explained by the presence of a mechanism assimilable to bile acid diarrhea (BAD), which is recognized in both humans [32,33] and dogs [34]. BAD occurs when excess primary bile acids enter the colon, leading to increased water secretion and motility, resulting in diarrhea. Some cases of non-responsive diarrhea with a good clinical response to cholestyramine treatment have been signaled in dogs [34], and in these cases we can hypothesize that the malabsorption of BAs is the primary cause of the diarrhea. In humans, the diagnosis of BAD is also associated with fatty liver disease and with gallstones [35]. In these cases, BAD is not linked to a defect in BA absorption. Instead, it is thought to stem from a primary defect in the feedback inhibition of hepatic BA synthesis, due to impaired secretion of the fibroblast growth factor 19 (FGF19) by the ileum [35]. In veterinary medicine, BAD has never been reported in dogs with CLDs. However, considering our results, we may suppose that CLD dogs presenting biliary tract involvement could also present clinical signs related to an excess of primary BAs in the intestinal lumen. This interesting finding requires further investigation. Conversely, we should also consider that diarrhea could have led to an increased rate of transit, ultimately reducing the time for the conversion of primary to secondary BAs and/or the reabsorption of primary BAs in the ileum.

This study has to be considered in view of its limitations. The number of patients enrolled is low and represented by various canine breeds, and a control group of healthy dogs was not recruited. Considering the broad interindividual variability, further studies involving a larger cohort of CLD dogs and a group of healthy dogs are necessary. Moreover, within the population, dogs with end-stage liver disease were not consistently represented. Another limitation may be represented by the enrollment of a heterogenous population of dogs with different kinds of CLD, which may have different ongoing pathogenetical mechanisms. Furthermore, GM assessment and hepatic histological assessment were not performed on each dog; thus, a definitive characterization of CLD according to an individual therapeutical and diagnostic process was not possible for each case. From a future perspective, it could be notable to investigate the potential correlation between the severity of the CLD and the alterations in fecal BA concentrations.

Also, as diet was not standardized, and diet can influence gut microbiota, which can subsequently influence the composition of BAs, we cannot exclude the possibility that our results may have been indirectly affected by the lack of diet standardization. Lastly, even though dogs with a history or ultrasonographic evidence of chronic enteropathy were not included in the present study, we cannot exclude the possibility that some mild and less detectable grades of concurrent enteropathy may have affected our results.

## 5. Conclusions

This was the first study investigating fecal BA profiling in dogs with CLD. The increase in fecal primary BAs (CA, CDCA) and total BAs in CLD dogs with biliary tract involvement is consistent with the trend observed in human patients with biliary tract disorders. BTD dogs also exhibited an increased prevalence of gastrointestinal signs and diarrhea, supporting the hypothesis of some pathological mechanisms assimilable to BAD. The observed alteration in fecal BAs in relation to biliary tract disease and serum ALT levels in CLD dogs could reflect imbalances of the liver–gut axis, with disrupted bile acid synthesis, altered enterohepatic circulation, and an alteration in the metabolic activity of the gut microbiota as possible pathogenetical pathways. Further evaluations involving GM and metabolomic assessment should be evaluated to better understand possible clinical implications of the impairment of BA metabolism and the potential role of BAD in canine CLD.

## Figures and Tables

**Table 1:** Descriptive statistics of serum hepatic enzymes, total bilirubin (Tot Bil), cholesterol (Chol), triglycerides (Trig), total protein (TP), and albumin (Alb) (expressed as the median and range) in CLD (chronic liver disease) dogs.

Biochemical Parameter	CLD Dogs (n = 46)	BTD (n = 28)	Non-BTD (n = 18)	Reference Range
ALP (U/L)	576.5 (84–8907)	745 (84–8907)	268 (86–1293)	45–250
GGT (U/L)	5.45 (1.1–375)	7.1 (1.1–375)	3.8 (1.4–30.6)	2–11
AST (U/L)	41 (18–324)	44 (18–324)	33 (18–97)	15–40
ALT (U/L)	121.5 (25–2384)	124 (37–2384)	109 (25–825)	20–70
Tot Bil (mg/dL)	0.22 (0.1–19.3)	0.22 (0.07–19.3)	0.22 (0.08–0.49)	0.07–0.3
TP (g/dL)	6.7 (4.4–9.8)	6.7 (4.4–9.8)	6.9 (5.2–8.5)	5.8–7.8
Alb (g/dL)	3.8 (2.7–5.4)	3.8 (2.7–5.4)	3.5 (3.1–4.5)	2.6–4.1
Chol (mg/dL)	308.5 (90–674)	355 (111–674)	259 (90–547)	120–280
Trig (mg/dL)	100 (45–1475)	105 (45–1475)	80 (45–277)	25–90

**Table 2:** Descriptive statistics of fecal bile acids (µmol/g): Cholic Acid (CA), Chenodeoxycholic Acid (CDCA), Ursodeoxycholic Acid (UDCA), Deoxycholic Acid (DCA), Lithocholic Acid (LCA), primary (CA + CDCA) and secondary BAs (UDCA + DCA + LCA), primary/secondary BA ratio, and total bile acids (expressed as median and range) in CLD, BTD, and non-BTD dogs.

Bile Acid	CLD Dogs (tot = 46)	BTD (n = 28)	Non-BTD (n = 18)	*p* Value
CDCA	151.32 (12.9–861.1)	237.4 (15.99–861.1)	100.3 (12.9–396)	0.003
UDCA	251.535 (32.5–1734.1)	233.5 (32.5–854.1)	262.7 (65–1734)	0.6
CA	268.398 (8.62–1305.4)	407 (27.76–1305)	112.7 (8.6–349.8)	0.0003
DCA	105.429 (7.5–2350.6)	199 (7.5–2351)	63.13 (9–857.2)	0.08
LCA	372.248 (46.9–1209.3)	299.1 (46.08–1209)	381.7 (52.9–1030)	0.7
Primary	450.281 (56.7–1822.7)	695.4 (91.2–1823)	289.4 (56.7–431.9)	<0.0001
Secondary	800.029 (168.5–3705.9)	800 (168.5–3705)	790.8 (250.1–3122)	0.3
Ratio P/S	0.572 (0.1–2.3)	0.831 (0.06–2.31)	0.3775 (0.1–0.9)	0.002
Total Bile Acids	1381 (361–4823)	1509 (481–4824)	1119 (361.5–3387)	0.005

## Data Availability

The complete data set is available upon reasonable request.

## Supplementary Materials

The following supporting information can be downloaded at: https://www.mdpi.com/article/10.3390/ani14213051/s1, In referral to the HPLC analysis of fecal bile acids, since all targeted compounds are detected at 254 nm, here we provide the limit of quantification (LOQ) and limit of detection (LOD) for each compound. Additionally, specifying the retention time for each compound. To provide other researchers with a point of reference for future studies, it would be beneficial to include at least one supplementary figure, such as a high-performance liquid chromatography (HPLC) chromatogram showing the retention time (RT), as a reference.
