# Supplementary material for: Fecal Bile Acids in Canine Chronic Liver Disease: Results from 46 Dogs
Source: Animals (Basel). 2024 Oct 22;14(21):3051. doi: 10.3390/ani14213051 (PMC11545594; doi:10.3390/ani14213051)
Supplement: Supplementary file 1 [file animals-14-03051-s001.zip › animals-3225534-supplementary.pdf]

In referral to the HPLC analysis of fecal bile acids, since all targeted compounds are detected at 254 nm, here we provide the limit of quantification (LOQ) and limit of detection (LOD) for each compound. Additionally, specifying the retention time for each compound. To provide other researchers with a point of reference for future studies, it would be beneficial to include at least one supplementary figure, such as a high-performance liquid chromatography (HPLC) chromatogram showing the retention time (RT), as a reference.

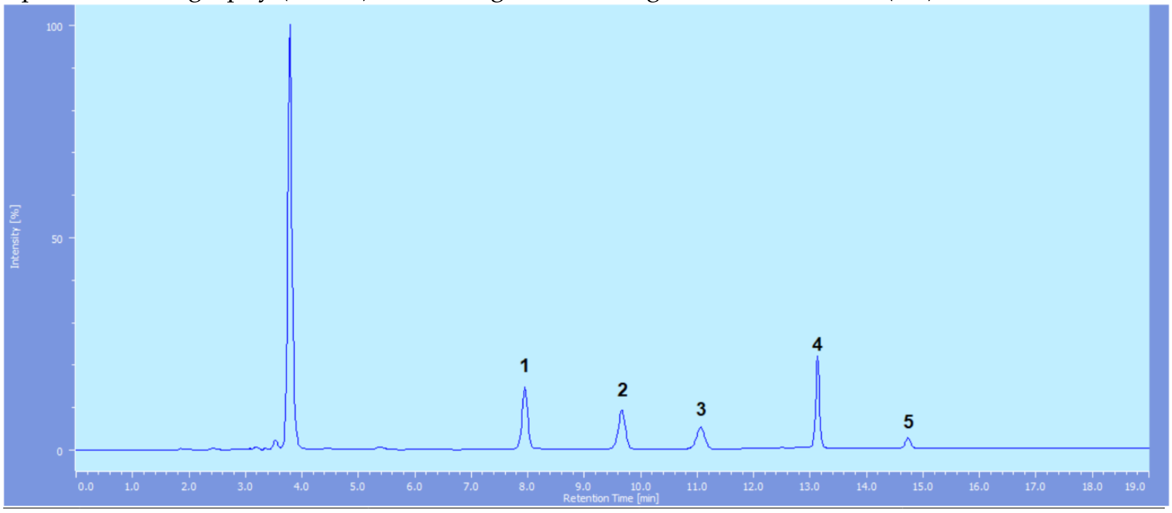

#	BA	Tr (mins)	LOD (µmol/g)	LOQ (µmol/g)
1	UDCA	8.03 ± 0.05	2.0	6.0
2	CA	9.75 ± 0.11	2.5	7.5
3	CDCA	11.13 ± 0.07	2.5	7.5
4	DCA	13.15 ± 0.08	2.0	6.0
5	LCA	14.75 ± 0.09	4.0	12.0
